# Artificial intelligence, machine learning, and deep learning in women’s health nursing

**DOI:** 10.4069/kjwhn.2020.03.11

**Published:** 2020-03-17

**Authors:** Geum Hee Jeong

**Affiliations:** School of Nursing and Research Institute in Nursing Science, Hallym University, Chuncheon, Korea

**Keywords:** Artificial intelligence, Big data, Computer neural networks, Deep learning, Nursing

## Abstract

Artificial intelligence (AI), which includes machine learning and deep learning has been introduced to nursing care in recent years. The present study reviews the following topics: the concepts of AI, machine learning, and deep learning; examples of AI-based nursing research; the necessity of education on AI in nursing schools; and the areas of nursing care where AI is useful. AI refers to an intelligent system consisting not of a human, but a machine. Machine learning refers to computers’ ability to learn without being explicitly programmed. Deep learning is a subset of machine learning that uses artificial neural networks consisting of multiple hidden layers. It is suggested that the educational curriculum should include big data, the concept of AI, algorithms and models of machine learning, the model of deep learning, and coding practice. The standard curriculum should be organized by the nursing society. An example of an area of nursing care where AI is useful is prenatal nursing interventions based on pregnant women’s nursing records and AI-based prediction of the risk of delivery according to pregnant women’s age. Nurses should be able to cope with the rapidly developing environment of nursing care influenced by AI and should understand how to apply AI in their field. It is time for Korean nurses to take steps to become familiar with AI in their research, education, and practice.

## Introduction

### Background/rationale

**Figure. 1.**
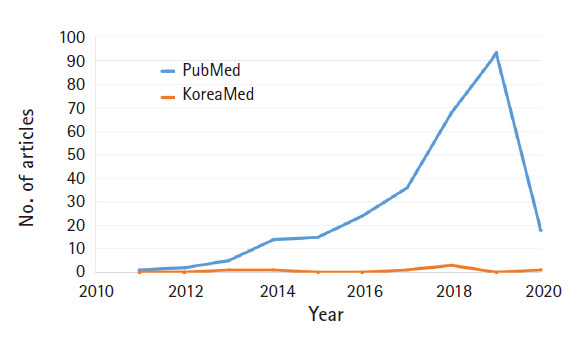
A number of articles on artificial intelligence, machine learning, or deep learning for nursing in PubMed (https://PubMed.gov) and KoreaMed (https://koreamed.org) according to year [cited 2020 March 2].

In recent years, artificial intelligence (AI) has been introduced into the field of nursing, and interest in its applications has grown explosively. As more specific forms of AI, machine learning and deep learning (eg, deep neural networks) have also been introduced. The number of articles in PubMed (https://PubMed.gov) on applications of AI, machine learning, and deep learning for nursing care has increased exponentially. In Korea, AI, machine learning, and deep learning have already been introduced into nursing care, although relatively few research articles have been published on this topic by domestic researchers. The results obtained using the search strings (“artificial intelligence” OR “machine learning” OR “deep neural network”) AND (nursing[Affiliation]) in PubMed and (“artificial intelligence” OR “machine learning” OR “deep neural network”) AND (nursing) in KoreaMed (https://koreamed.org) are presented in Figure 1. However, relatively little of this content focuses on nurses’ jobs. Nonetheless, nurses are already living in an AI environment, making it necessary to understand how to apply AI to nursing.

### Objectives

This article addresses the following questions. First, what are AI, machine learning, and deep learning? Second, in which nursing research areas has AI been adopted? Third, is it necessary to introduce education on AI into the nursing curriculum? Fourth, which aspects of nursing care for women’s health are promising targets for the application of AI?

## What are AI, machine learning, and deep learning?

In the Merriam-Webster Dictionary, AI is defined as follows [1]:

1. a branch of computer science dealing with the simulation of intelligent behavior in computers

2. the capability of a machine to imitate intelligent human behavior

The definition of AI in Wikipedia is as follows:

In computer science, artificial intelligence (AI), sometimes called machine intelligence, is *intelligence demonstrated by machines*, in contrast to the natural intelligence displayed by humans. Leading AI textbooks define the field as the study of “intelligent agents”; any device that perceives its environment and takes actions that maximize its chance of successfully achieving its goals. Colloquially, the term “artificial intelligence” is often used to describe machines (or computers) that mimic “cognitive” functions that humans associate with the human mind, such as “learning” and “problem-solving” [2].

**Figure. 2.**
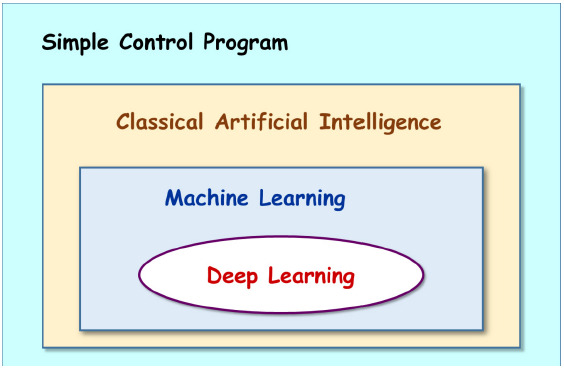
Diagram of the relationship of terms for the level of artificial intelligence.

An accessible way to understand AI is to present it in terms of levels: The first level of AI is a simple control program directly controlled by the operator. The second level is classical AI with multiple patterns and an enormous number of input-output mappings; such systems are used for searching and making estimations, and as knowledge databases. The third level is machine learning-based AI. The fourth level is deep learning-based AI (Figure 2).

Machine learning is a field of study that gives computers the ability to learn without being explicitly programmed, according to Samuel’s definition in 1959 [3]. Wikipedia describes machine learning as “the scientific study of algorithms and statistical models that computer systems use to perform a specific task without using explicit instructions. It relies on patterns and inference instead. It is seen as a subset of AI. Machine learning algorithms build a mathematical model based on sample data, known as ‘training data’, to make predictions or decisions without being explicitly programmed to perform the task” [4]. Data mining is an example of machine learning. Machine learning is executed based on the data, not on experts’ opinions. There are several types of machine learning models, including artificial neural networks, decision trees, support vector machines, regression analysis, Bayesian networks, and genetic algorithms.

**Figure. 3.**
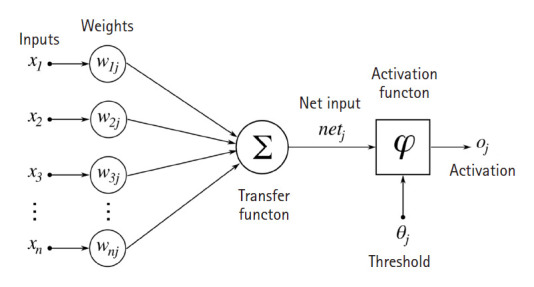
Diagram of the artificial neuron (deep learning). Source: created by Chrislb (CC BY-SA 3.0, https://commons.wikimedia.org/w/index.php?curid=224555) [cited 2020 Mar 3].

Deep learning is a model of machine learning using artificial neural networks that consist of multiple hidden layers, which is why these neural networks are known as “deep” neural networks and the framework is known as “deep learning.” Deep learning can be implemented as shown in Figure 3, where the input value (*x*) is multiplied by the weight (*w*), added up, and then output through specific functions such as the net input function and activation function. Within the broader category of machine learning models, deep learning can be thought of as an analytical method using neural networks. Deep learning is useful if relevant data exist, but problems of interest cannot be solved by extant methods. For example, deep learning is used for the game of Go, face recognition, object recognition, voice recognition, and translation [5].

## In which nursing research areas has AI been adopted?

Through a search of the PubMed and KoreaMed databases, it was found that there are still relatively few studies on AI in the field of nursing. It was also difficult to find relevant research in the field of women’s health nursing.

Below are some examples of nursing studies on AI conducted outside of Korea:

First, a chatbot was developed for mental health caregiving. The researchers described it as follows: “One mental health chatbot, or psychological AI service named Tess, has been used to deliver on-demand support for caregiving professionals, patients, and family caregivers at a non-profit organization. This low-cost, user-friendly, and highly customizable service allows emotional support to be scaled to thousands of people at a single time.” This is an example of integration of a smart chatbot and AI to help caregivers [6].

Second, a natural language processing system was developed that identifies relationships of medications with adverse drug events. A corpus of 505 de-identified clinical notes was trained with a recurrent convolutional neural network and showed excellent performance. This learning system can be used to extract relationships between medications and adverse drug events [7].

Third, a sensor placed on the floor was developed for monitoring elderly individuals in a nursing home, since monitoring the activities of elderly individuals is necessary to check on their health. The sensor was installed on the floor and analyzed their gaits. Deep learning was not able to recognize elderly individuals, but it achieved very high accuracy for classifying the elderly and medical staff [8].

Some examples of nursing studies on AI from Korea are described below:

First, a tool was developed to predict health-related quality of life. Secondary data on 716 individuals extracted from the Korea National Health and Nutrition Examination Survey from 2008 to 2010 were analyzed to identify factors affecting health-related quality of life. Analytical tools, including support vector machine learning, found the following five key factors that predicted health-related quality of life: income, chronic diseases, depression, discomfort, and perceived health status [9].

Second, a nursing diagnosis system was developed. Using a back-propagation neural network, a nursing diagnosis system was tested. The network was composed of three layers for training: one input layer of data from 119 patients; one output layer for the nursing diagnosis; and one hidden layer including six nodes of the hidden layer, a momentum of 0.5, and a learning coefficient of 0.5. The coincidence ratio was 0.93, with 20,000 rounds of learning. This system demonstrated the possibility of applying neural networks to nursing diagnoses [10].

Third, a system was developed for classifying data on adherence to medication. A support vector machine (a machine learning method useful for data classification) was applied to identify factors predicting adherence to medication in heart failure patients. After analysis of 11 variables from 76 patients, two models were suggested: one with five predictors, and the other with seven predictors. Both models showed an accuracy of 77.6%. This machine learning method helped to stratify patients [11].

Fourth, a triage system for the emergency department was developed. Through an emergency department triage system based on machine learning and the initial nursing assessment, an efficient system to predict adverse clinical outcomes could be created. This new system was more efficient than the previous traditional system [12].

## Is it necessary to introduce education on AI into the nursing curriculum?

It is difficult for nursing faculty members and nursing school leaders to decide whether AI, machine learning, and deep learning should be introduced into the curriculum of nursing education. Although the curriculum has limited hours, AI should be introduced to keep up with this new paradigm that affects both society as a whole and research. Since there currently is no specific curriculum dealing with AI for nursing students, AI should be discussed in nursing schools. To achieve these goals, professional associations dedicated to nursing education or related organizations, such as the Korean Accreditation Board of Nursing Education, should consider adopting a new curriculum on AI.

Paranjape et al. [13] suggested the following content for education on AI in medical schools: the fundamentals of AI, electronic hospital records and data, application of AI to clinical decision-making, and ethical and legal aspects of AI. I suggest the following content for an AI curriculum in nursing education: nursing data as big data, the concept of AI, algorithms, models of machine learning, and the model of deep learning. Additionally, the curriculum should include coding practice with Python, TensorFlow, and Keras. This content can be incorporated throughout nursing education, from the undergraduate level to graduate coursework, depending on the preparedness of the teaching faculty. A more specific and well-woven curriculum should be developed for each grade. The application of AI will certainly be beneficial; therefore, professors need to understand the principles of AI and prepare to integrate AI into the nursing curriculum.

## Which aspects of nursing care for women’s health are promising targets for the application of AI?

AI can be applied to data relating to any topic of interest. Some examples include prenatal nursing interventions according to pregnant women’s nursing records, prediction of the risk of delivery according to pregnant women’s age, prediction of postpartum depression, risk of breast cancer before and after menopause, the needs for nursing care among elderly women with physical disabilities, and trends in women’s health nursing research and practice. Because there are so many topics for which AI, machine learning, and deep learning are promising approaches, any topic of interest in the field of women's health nursing care could be a target for AI applications if nurses are interested in exploring the specific outputs that result from certain data.

## Conclusion

AI, including machine learning and deep learning, is an inevitable trend that is currently shaping the environment of nursing research, education, and practice, and will continue to do so in future decades. As nurses, we should be able to understand the principles of AI and apply AI in a broad range of fields. Regarding AI as a statistical method may make it easier to tackle. After understanding the technology and concepts of AI, the essential material is data, especially big data for deep learning. Therefore, data manipulation based on a careful understanding of the data is also an essential competency for working with AI. If we can manage data appropriately and enter high-quality data during the analysis process, it will be easier to obtain better results from AI-based studies. A standard AI curriculum should be established and introduced in nursing education as soon as possible. Each nursing school can modify the standard curriculum according to the faculty’s competency and capacity of facilities. In short, it is time for Korean nurses to adopt AI in their research, education, and practice.
